# T-LAK cell-originated protein kinase presents a novel therapeutic target in *FLT3*-ITD mutated acute myeloid leukemia

**DOI:** 10.18632/oncotarget.5418

**Published:** 2015-10-02

**Authors:** Houda Alachkar, Martin Mutonga, Gregory Malnassy, Jae-Hyun Park, Noreen Fulton, Alex Woods, Liping Meng, Justin Kline, Gordana Raca, Olatoyosi Odenike, Naofumi Takamatsu, Takashi Miyamoto, Yo Matsuo, Wendy Stock, Yusuke Nakamura

**Affiliations:** ^1^ Department of Medicine, Section of Hematology/Oncology, University of Chicago, Chicago, IL, USA; ^2^ OncoTherapy Science, Inc., Kanagawa, Japan

**Keywords:** AML, FLT3-ITD, TOPK, CEBPA, kinase inhibitor

## Abstract

Gain-of-function mutations of *FLT3* (*FLT3*-ITD), comprises up to 30% of normal karyotype acute myeloid leukemia (AML) and is associated with an adverse prognosis. Current FLT3 kinase inhibitors have been tested extensively, but have not yet resulted in a survival benefit and novel therapies are awaited. Here we show that T-LAK cell-originated protein kinase (TOPK), a mitotic kinase highly expressed in and correlated with more aggressive phenotype in several types of cancer, is expressed in AML but not in normal CD34+ cells and that *TOPK* knockdown decreased cell viability and induced apoptosis. Treatment of AML cells with TOPK inhibitor (OTS514) resulted in a dose-dependent decrease in cell viability with lower IC_50_ in *FLT3*-mutated cells, including blasts obtained from patients relapsed after FLT3-inhibitor treatment. Using a MV4-11-engrafted mouse model, we found that mice treated with 7.5 mg/kg IV daily for 3 weeks survived significantly longer than vehicle treated mice (median survival 46 vs 29 days, *P* < 0.001). Importantly, we identified TOPK as a *FLT3*-ITD and CEBPA regulated kinase, and that modulating TOPK expression or activity resulted in significant decrease of FLT3 expression and CEBPA phosphorylation. Thus, targeting TOPK in *FLT3*-ITD AML represents a novel therapeutic approach for this adverse risk subset of AML.

## INTRODUCTION

Gain-of-function mutations of the tyrosine kinase (TK) receptor encoding FMS-like tyrosine kinase-3 (FLT3) have been observed in approximately 30% of cytogenetically normal acute myeloid leukemia (AML). Of the *FLT3* mutations, the internal tandem duplication (*FLT3*-ITD) is associated with poorer outcome [1–6]. The *FLT3*-ITD and overexpression of *FLT3* constitutively activate several pathways like MAPK/ERK, PI3K/AKT, NF-κB and STAT5 [7–10], and repress important transcription factors involved in myeloid differentiation, PU.1 and CEBPA [7, 11, 12]. Thus, *FLT3*-ITD mutation results in both increased cell proliferation and block of myeloid differentiation [12, 13]. In addition, overexpression of the FLT3-wt receptor and its ligand (FL) occurs in a high proportion of AML and activates the autocrine stimulatory loop contributing to the pathogenesis and aggressiveness of the disease [14, 15]. Therefore, compounds that inhibit both mutant and overexpressed wild type FLT3 in AML may provide a more effective therapeutic strategy than compounds that only target the kinase activity of the mutant FLT3. Indeed, emerging small molecule inhibitor compounds have been shown to interfere with the aberrant FLT3 TK activity and lead to arrest of leukemia growth [4]. Unfortunately the clinical impact of these compounds has not yet fulfilled the promise, possibly due to their insufficient inhibition of FLT3, lack of specificity, and the rapid emergence of resistance mechanisms in addition to pharmacokinetic limitations [16–19]. A recent study indicates that an ERK signaling pathway is likely to play a role in the mechanism of resistance to FLT3 inhibition conferred by stroma and FL ligand [20]. Indeed, some alternative approaches for targeting FLT3-mutated AML have been focused on targeting downstream pathways (ERK1/2, CDK1, STAT5) that are activated by *FLT3*-ITD [11, 12, 21, 22].

T-LAK cell–originated protein kinase (TOPK) is a serine-threonine kinase that is highly expressed in and correlated with a more aggressive phenotype in several types of cancer such as breast cancer, Ewing sarcoma, and colorectal cancer [23–31]. TOPK is hardly detectable in normal tissues except the testis and fetal tissues [32]. TOPK was found to be involved in cancer cell mitosis and proliferation [23, 26, 33].

TOPK belongs to the mitogen-activated protein kinase kinase (MAPKK) family, a major part of the RAS/RAF/MEK/ERK signaling axis. The latter is known to be activated in AML and targeting the different components of this pathway has been extensively investigated [34, 35]. Furthermore, a positive feedback loop between TOPK and ERK2 has been identified to promote development of colorectal cancer [25]. TOPK was also reported to increase cell migration by modulating a PI3K/PTEN/AKT-dependent signaling pathway [36]. Recently, TOPK was found to be upregulated in prostate cancer and associated with tumor invasiveness likely via activating MMPs and β-catenin pathway [37]. In addition, CDK1/Cyclin B1 activates TOPK during mitosis, which subsequently promotes cytokinesis [33, 38, 39]. Although TOPK was also reported to be up-regulated in a variety of hematologic malignancies including AML [40], TOPK expression and function in AML has not been fully elucidated.

Because of the possible interplay between TOPK and other molecular targets known to be involved in leukemia such as ERK1/2, CDK1, and CyclinB1, we hypothesized that TOPK might play some role in AML and therefore may serve as a therapeutic target in this disease. Here we demonstrate that targeting TOPK expression or kinase activity results in suppression of *FLT3* expression and inhibition of CEBPA phosphorylation leading to enhanced myeloid differentiation and remarkable *in vitro* and *in vivo* anti-leukemia activity, particularly in *FLT3*-ITD AML. We also identified a mechanistic network by which TOPK is deregulated in AML that involves both *FLT3* and *CEBPA*, genes often mutated in AML and that play a central role in leukemogenesis. Therefore we conclude that TOPK represents a novel therapeutic target for *FLT3*-ITD AML.

## RESULTS

### TOPK is expressed in AML cell lines and primary AML blasts

To determine the level of TOPK expression in AML, we assessed, by western blot analysis, the protein level in 11 AML cell lines representative of the different cytogenetic and molecular subsets of AML (Supplementary Table S1), as well as in primary leukemia blasts from 12 patients with AML. TOPK protein was highly expressed in the majority of AML cell lines, and in 7 of 12 AML primary blasts; however TOPK was not detected in peripheral CD34+ mobilized cells from healthy donors (Supplementary Figure S1A and S1B).

### TOPK knock-down results in decreased cell viability and increased apoptosis

**Figure 1:**
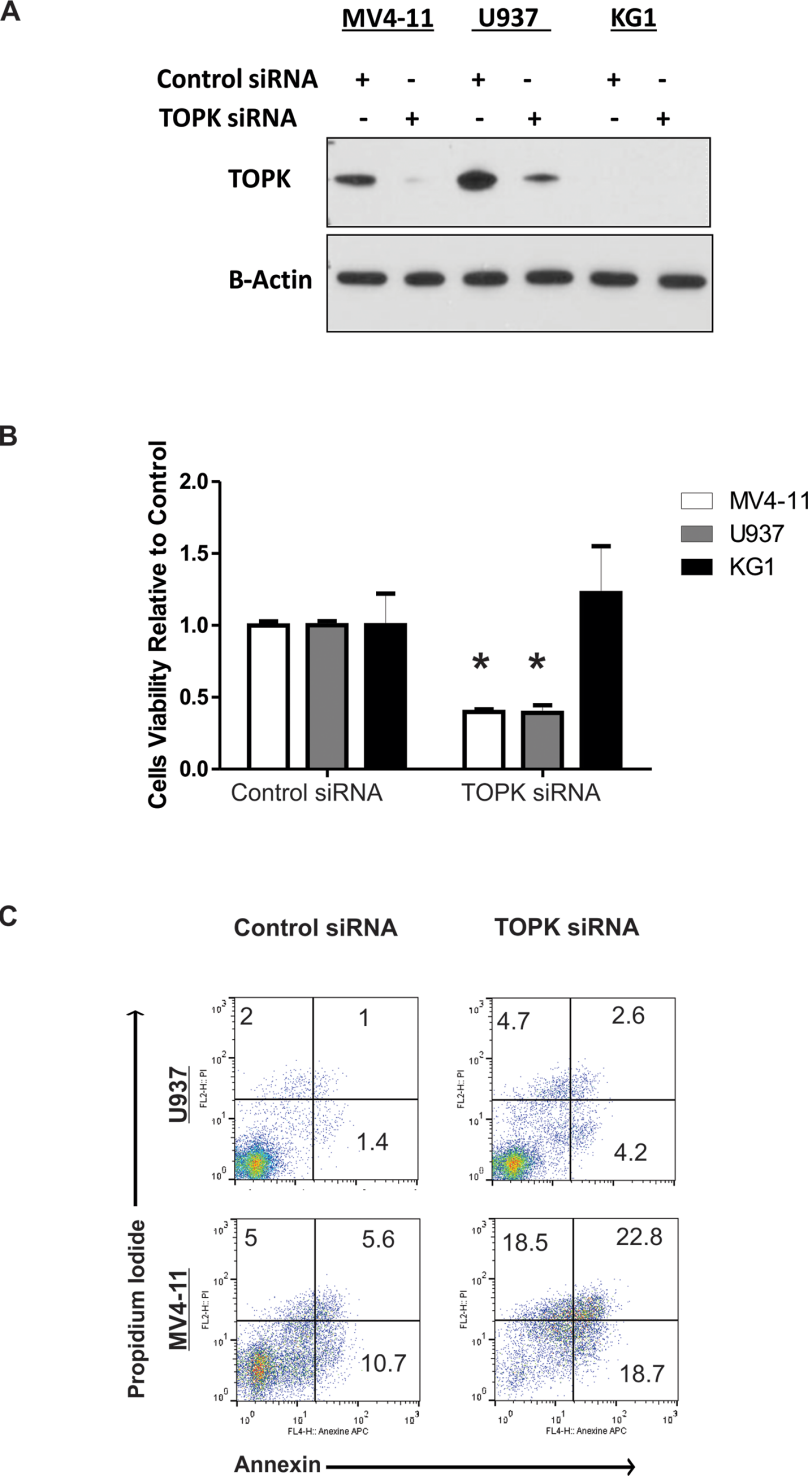
TOPK Knock-down decreases cell viability and induces apoptosis MV4-11, U937 and KG1 cells were transfected with TOPK siRNA or control siRNA; **A.** western blot was performed to measure TOPK protein level. **B.** Viability assay was performed 48 hours following transfection. **C.** Apoptosis assay was performed using annexin and PI staining in MV4-11 and U937 cells 48 hours following transfection. Data are presented as Mean ± SEM, *P* values were calculated using Student's *t*-test (*P < 0.05).

To investigate TOPK as a potential therapeutic target in AML, we utilized a loss of function approach in two AML cell lines that express relatively high levels of TOPK protein (MV4-11, U937), and in the KG1 cells that express a very low level of TOPK, as a control. TOPK knock-down (Figure 1A) resulted in significant decrease (approximately 60%; *P* < 0.001) in cell viability in MV4-11 and U937 cells, while no significant effect was observed in KG1 cells (Figure 1B). In addition, annexin/PI staining assessed by flow cytometry showed that TOPK knock-down resulted in a dramatic increase in apoptosis in MV4-11 cells; less effect on apoptosis was observed in U937 cells (Figure 1C). We also validated this effect in MOLM13 cells that express high level of TOPK (Supplementary Figure S2).

### TOPK inhibitor OTS514 exhibits cytotoxic activity in AML cells but not in normal CD34+ cells

**Figure 2:**
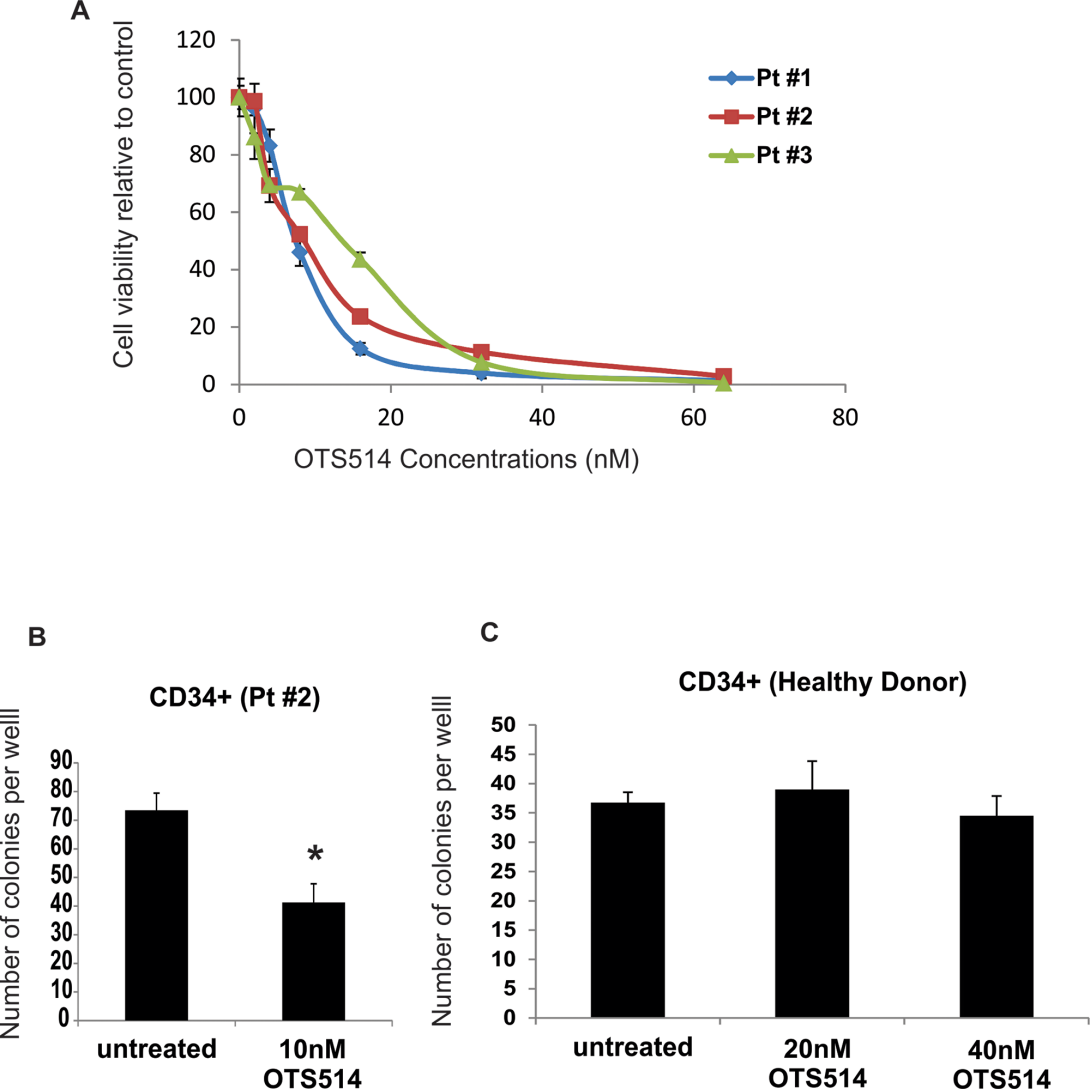
TOPK inhibitor inhibits colony formation in leukemia but not normal CD34+ cells AML blasts were treated with TOPK inhibitor OTS514. **A.** Viability assay was performed in AML blasts obtained from three AML patients 48 hours following treatment with increasing concentration of OTS514. **B.** Colony forming assay was performed in sorted CD34+ cells obtained from AML patient and treated with 10 nM of OTS514. **C.** Colony forming assay was performed in CD34+ cells obtained from healthy donor and treated with 20 and 40 nM of OTS514. Data are presented as Mean ± SEM, *P* values were calculated using Student's *t*-test (*P < 0.05).

Having shown that TOPK knock-down resulted in enhancement of apoptosis and decrease in cell viability, we then examined whether targeting TOPK kinase activity with a recently developed TOPK inhibitor OTS514 [41] would result in a cytotoxic effect in AML cells. We treated primary blasts obtained from 3 patients with AML with different concentrations of OTS514, and found a dose dependent decrease in cell viability in all three samples, with an IC_50_ that ranged from 10–20 nM (Figure 2A). To further investigate the cytotoxic effect of OTS514 in AML, CD34+ cells obtained from a patient with AML (AML-CD34+) and those from a healthy donor (normal-CD34+) were treated with OTS514, and assessed for colony forming ability. We found a significant decrease in the number of colonies per well in AML-CD34+ cells treated with 10 nM of OTS514 compared to untreated cells (41 vs 73, *P* = 0.01) (Figure 2B). In contrast, no effect was observed following 20 nM or 40 nM of OTS514 treatment of CD34+ cells obtained from healthy donors (39 vs 36, *P* = 0.67; and 34 vs 36 *P* = 0.57) (Figure 2C).

### TOPK inhibitor exhibits preferential anti-leukemia activity in AML with *FLT3* mutation

**Figure 3:**
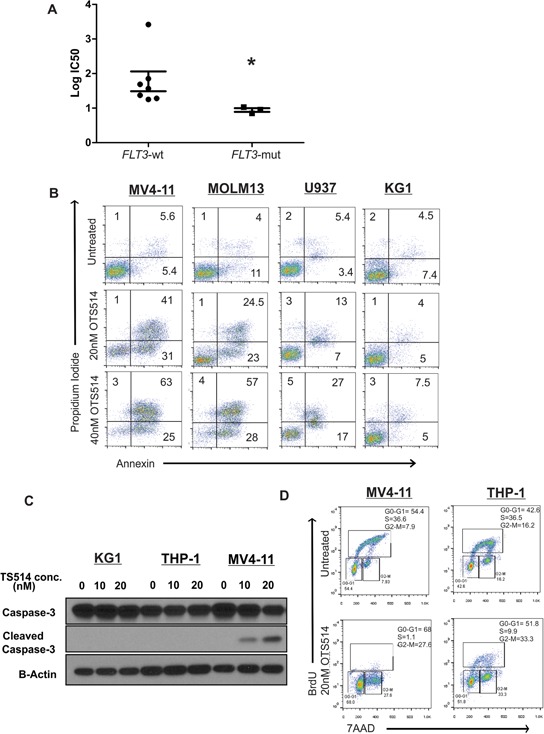
TOPK inhibitor exhibits preferential anti-leukemia activity in *FLT3* mutated AML **A.** AML cell lines (*n* = 10) were treated with increasing concentration of TOPK inhibitor OTS514, and viability assay was performed 48 hours post-treatment, calculated IC50 were compared between *FLT3*-mut cell lines and *FLT3*-wt cell lines. **B.** MV4-11, MOLM13, U937 and KG1 cells were treated with 20 and 40 nM of OTS514 and assessed for apoptotic cells by annexin and PI staining. **C.** MV4-11, THP-1 and KG1 cells were treated with 20 nM OTS514 and cleaved caspase 3 levels were assessed by western blot and **D.** cell cycle analysis was performed using BrdU and 7AAD staining. Data are presented as Mean ± SEM, *P* values were calculated using Mann-Whitney *U* test (*P < 0.05).

In order to examine whether a specific subset(s) of AML is more or less sensitive to TOPK inhibition, we selected 10 AML cell lines that represent the different molecular and cytogenetic aberrations (Supplementary Table S1), and treated these cell lines with different concentrations of OTS514. Variable sensitivity to the TOPK inhibitor among the different cell lines was observed. Interestingly cell lines that carried *FLT3* mutations (MV4-11, MOLM13 and KOCL-48) revealed significantly higher sensitivity to OTS514 than other cell lines (Mann-Whitney *U* test; *P* = 0.016) (Figures 3A and Supplementary Figure S3).

We further confirmed the activity of this compound by annexin/PI staining in MV4-11 and MOLM13 cell lines (carrying *FLT3*-ITD mutation and expressing relatively high level of TOPK), U937 (*FLT3*-negative cell line expressing TOPK), and KG1 cells (*FLT3*-wt with very low expression of TOPK). When cells were treated with 40 nM of OTS514 for 48 hours, we observed an 80% and 70% increase in apoptotic cell population in MV4-11 and MOLM13 cells, respectively, but only a 40% and 10% increase in U937 and KG1 cells, respectively (Figure 3B). Consistent with these findings, cleaved caspase 3 was observed in MV4-11 cells but not in THP-1 (*FLT3*-wt with high expression of TOPK) or KG1, when these cells were treated under the same dose and duration (Figure 3C). To further validate that *FLT3*-ITD mutated AML cells are more sensitive to OTS514, we evaluated cell cycle kinetics in MV4-11 and THP-1 cells treated with 20 nM of OTS514 compared with untreated cells. MV4-11 cells showed a drastic decrease (~98%; *P* = 0.003) in the S phase by 24- and 48-hour treatment; while we observed 74% and 27% decrease in the S phase in THP-1 cells (*P* < 0.001 and *P* = 0.02), respectively (Figure 3D and Supplementary Figure S4).

**Figure 4:**
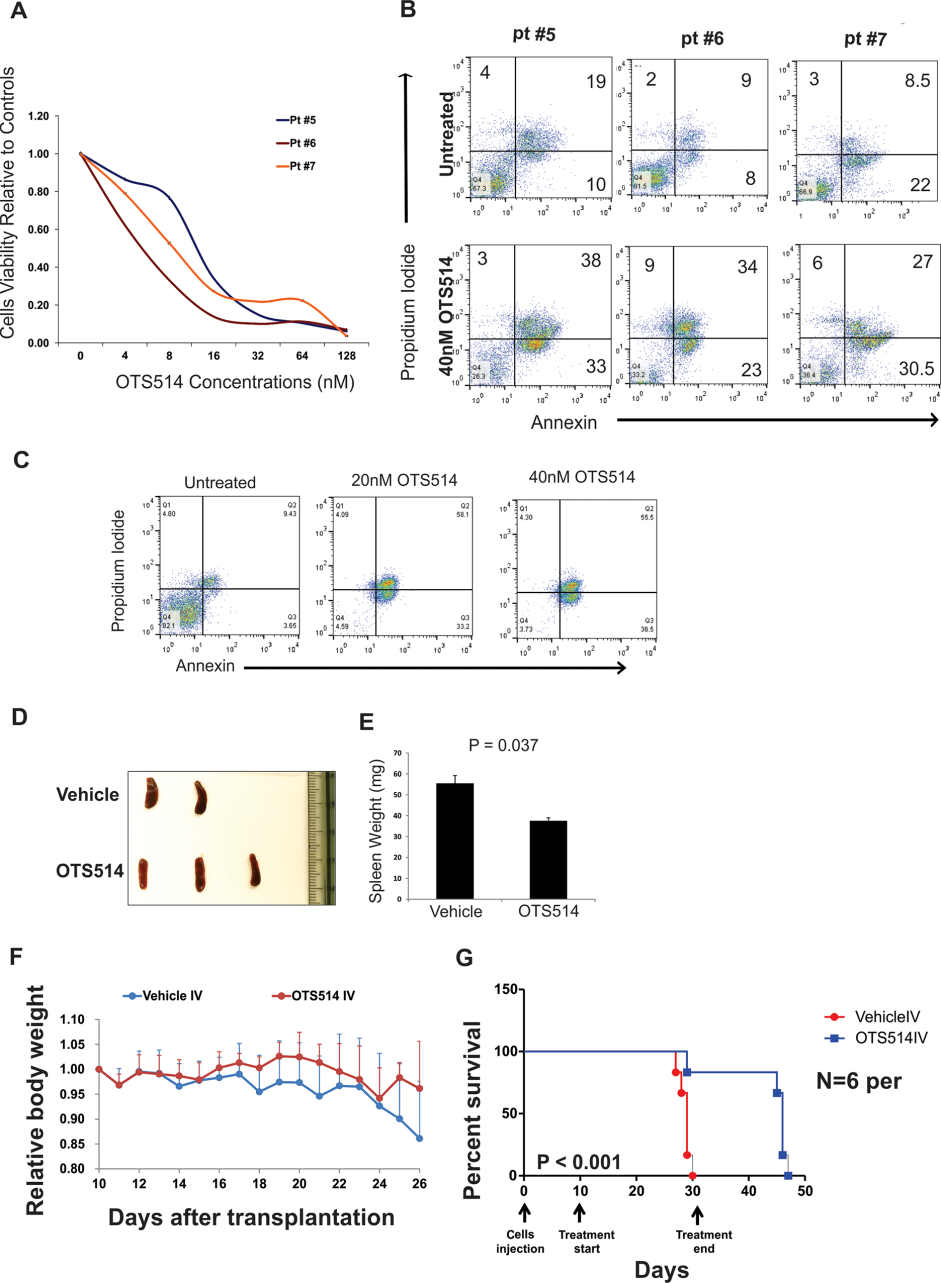
TOPK inhibitor exhibits anti-leukemia activity in *FLT3* mutated AML blasts and in MV4-11 murine model **A.** Blasts obtained from three AML patients with *FLT3*-ITD mutations and relapsed following AC220 clinical trial, cells were treated with increasing concentration of OTS514 and viability assay was performed 48 hours later. **B.** Apoptosis was assessed by annexin and PI staining in blasts obtained from three AML patients with *FLT3*-ITD mutation **C.** Blasts from AML patient with *FLT3*-ITD mutation were treated with 20 and 40 nM of OTS514 and apoptotic cells were assessed by annexin and PI staining 5 days later. **D.** Image of spleens obtained from vehicle and OTS514 treated mice. **E.** Quantitative analysis of spleen weights (Mean ± SEM). **F.** Mean relative mouse body weight ± SD (*N* = 6 mice per group) in comparison with the body weight just before the administration. **G.** Survival analysis of OTS514-treated leukemic mice (*N* = 6) compared with the vehicle-treated controls (*N* = 6) (*P* < 0.001).

The anti-leukemia activity of TOPK inhibition was also validated in primary blast cells obtained from three patients with AML with *FLT3*-ITD mutation who had relapsed after treatment with a potent FLT3 inhibitor (AC220 [quizartinib]) (Supplementary Table S2, Figure 4A). Apoptosis measured by annexin/PI staining was significantly increased (50–80%) in primary blasts treated with 40 nM of OTS514 (Figure 4B). Primary blasts obtained from an additional patient were treated with one dose of either 20 or 40 nM of OTS514, and leukemia cells were completely eradicated by apoptosis five days later as shown in Figure 4C.

### TOPK inhibitor exhibits *in vivo* anti-leukemia activity in a MV4-11 engraft NSG mouse model

Having demonstrated the high preferential activity of TOPK inhibitor OTS514 in AML cell lines and primary blasts with *FLT3*-ITD mutation, we attempted to examine the *in vivo* activity of this compound using a previously established *FLT3*-ITD engraft murine model [42]. For pharmacodynamics study, five mice were transplanted with 1.0 × 10^5^ of spleen cells obtained from MV4-11 transplanted NSG mice. Starting on day 13 after engraftment, mice were treated with OTS514 (*N* = 3, 7.5 mg/kg IV) or vehicle (*N* = 2, same volume IV) daily for 4 days. Mice were sacrificed 24 hours after the last dose, and spleens were obtained and weighed. Spleens obtained from mice treated with OTS514 showed significantly lower weight than spleens obtained from vehicle treated mice (*P* = 0.037; Figure 4D and 4E). For survival analysis, six mice were used for each with of the treatment groups. Spleen cells (1.0 × 10^5^) from the fifth generation of MV4-11 transplanted NSG mice were injected into NSG mice via the tail vein. Treatment with OTS514 (7.5 mg/kg IV) or vehicle (same volume IV) was given daily for 3 weeks starting 10 days after engraftment. Mice were weighed daily and checked for signs of dehydration, discomfort or toxicity. Although one mouse in the treatment group died after the 19^th^ injection, the bone marrow of that mouse had a normal appearance with red color unlike the white bone marrow (leukemia infiltration) of two other vehicle treated mice that died on the same day. This suggests that the death of the OTS514-treated mouse was more likely due to toxicity rather than leukemia progression (Supplementary Figure S5). We did not observe any decrease in body weight in mice treated with OTS514 (Figure 4F). Importantly, we found that mice treated with 7.5 mg/kg IV every day for 3 weeks survived significantly longer than the control mice (median survival 46 days vs 29 days, *P* < 0.001; Figure 4G).

### Targeting TOPK downregulates *FLT3* expression in AML cells

**Figure 5:**
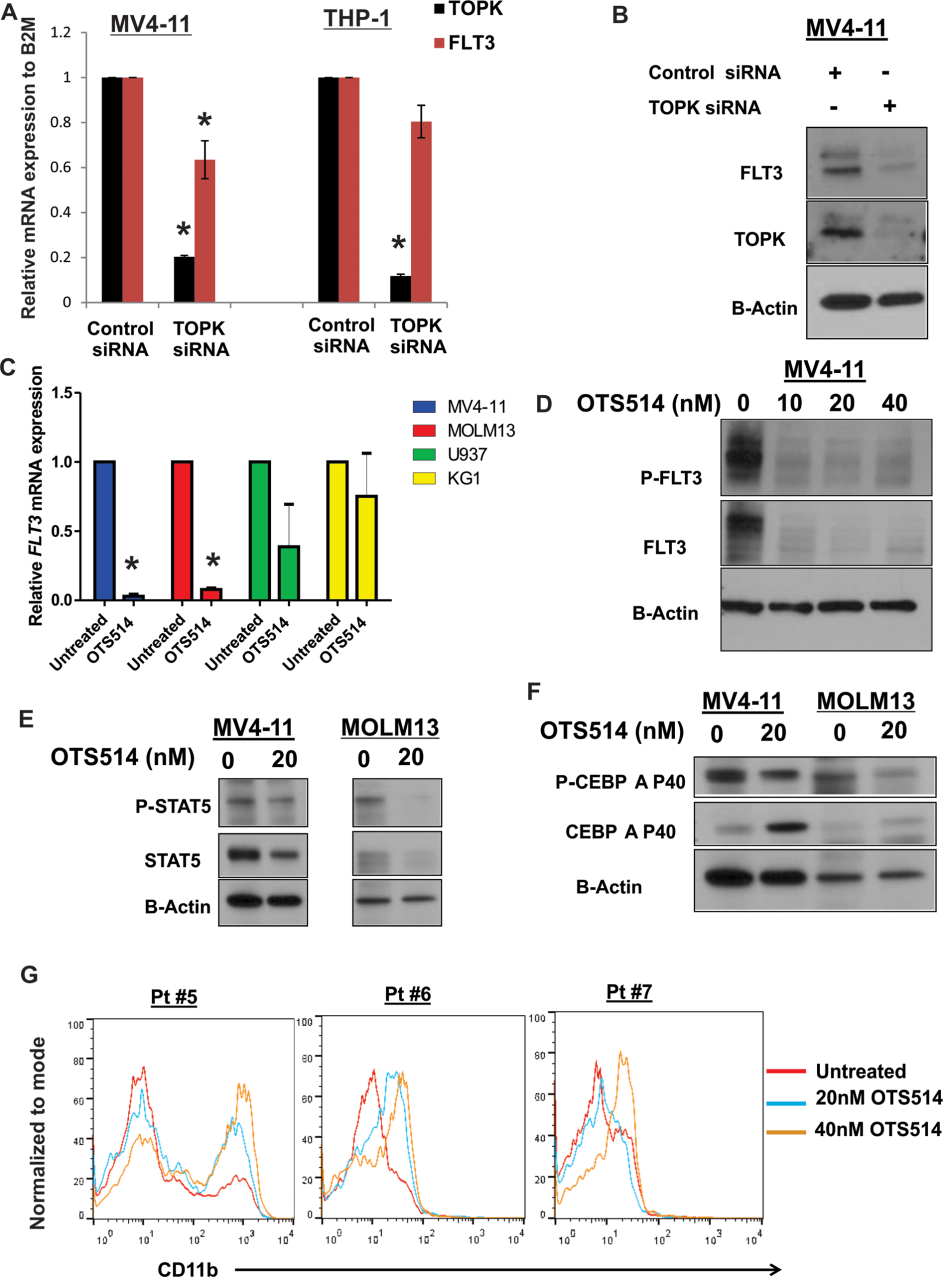
Targeting TOPK downregulates FLT3 expression, decreases CEBPA phosphorylation and induces myeloid differentiation in AML cells **A.** MV4-11 and THP-1 cells were transfected with TOPK siRNA or control siRNA and *FLT3* mRNA level was measured 48 hours later by qRT-PCR, and **B.** FLT3 protein levels were measured by western blot. **C.** MV4-11, MOLM13, U937 and KG1 cells were treated with 10 nM of OTS514 and *FLT3* mRNA levels were measured by qRT-PCR 18 hours later. **D.** MV4-11 cells were treated with 10, 20 and 40 nM of OTS514 and P-FLT3 and total FLT3 protein level was assessed by western blot. **E.** MV4-11 and MOLM13 cells were treated with 20 nM of OTS514, P-STAT5 and totalSTAT5 were assessed by western blot. **F.** MV4-11 and MOLM13 cells were treated with 20 nM of OTS514, P-CEBPA and total CEBPA were assessed by western blot 18 hours later. **G.** CD11b expression was assessed by flow cytometry in blasts obtained from three AML patients with *FLT3*-ITD mutation following treatment with 20 and 40 nM of OTS514 (data were normalized to mode fluorescence intensity). Data are presented as Mean ± SEM, *P* values were calculated using Student's *t*-test (*p < 0.05).

To provide insights into the molecular mechanism by which TOPK inhibition results in stronger growth suppression in *FLT3*-ITD mutated AML cells in comparison to *FLT3*-wt AML cells, we performed gene expression microarray analysis in *FLT3*-ITD mutated MV4-11 cells transfected with *TOPK*-siRNA or treated with OTS514, compared with control MV4-11 cells. Genes involved in cell cycle control such as CCNB1, CHECK1 and CDC25A were among those most strongly downregulated. Interestingly, *FLT3* was also one of the genes that were down-regulated in MV4-11 cells treated with OTS514 as well as those transfected with *TOPK*-siRNA, compared with the respective MV4-11 control cells. We validated these findings by qRT-PCR (*P* = 0.01; Figure 5A) and also demonstrated that TOPK knockdown reduced FLT3 protein level in MV4-11 cells (Figure 5B). OTS514 treatment also resulted in downregulation of *FLT3* transcription in *FLT3*-ITD mutated cells (Figure 5C). In addition, we found that total and phospho-FLT3 protein levels were significantly depleted in MV4-11 cells treated with the TOPK inhibitor (Figure 5D). Likewise, we found that protein and phosphorylation levels of STAT5, a FLT3-ITD downstream target, were significantly reduced in MV4-11 and MOLM13 cells that were treated with 20 nM of OTS514 (Figure 5E). To further confirm that the reduction of FLT3 protein by the TOPK inhibitor in FLT3-ITD cells was caused by inhibition of *FLT3* transcription rather than enhancement of protein degradation, we assessed FLT3 protein stability following OTS514 treatment and found no significant difference in the FLT3 protein level between MV4-11 cells co-treated with cyclohexamide (CHX; an inhibitor of protein translation) and 20 nM of OTS514 and those treated with CHX alone (Supplementary Figure S6). Furthermore, the activity of TOPK and FLT3 kinase were measured after incubation of each recombinant protein with increasing concentrations of OTS514. OTS514 at a concentration of 1.2 μM resulted in more than a 10 folds higher kinase inhibition of TOPK than that of FLT3 (Supplementary Figure S7).

### Targeting TOPK decreases CEBPA phosphorylation and induces myeloid differentiation

Based on a recent report implicating CEBPA as a key regulator of *FLT3* expression in AML [43] as well as previous studies suggesting that promotion of granulocytic differentiation by CEBPA is inhibited in *FLT3*-ITD AML via ERK1/2- or CDK1-mediated phosphorylation of serine-21 [22, 44], we examined whether CEBPA phosphorylation is modulated by TOPK activity. To test this, we first examined CEBPA phosphorylation following TOPK inhibitor treatment in MV4-11 and MOLM13 cells. Interestingly, p-CEBPA was decreased by 24-hour treatment of 20 nM of OTS514 (Figure 5F). To further confirm that CEBPA phosphorylation is modulated by TOPK activity, we utilized a loss of function approach. MV4-11 cells transfected with *TOPK*-siRNA showed a decrease in p-CEBPA compared with cells transfected with control-siRNA (Supplementary Figure S8A). Similarly, cells transfected with FLT3 siRNA showed a decrease in p-CEBPA levels (Supplementary Figure S8B). Therefore, we speculated that OTS514 might enhance myeloid differentiation in *FLT3*-ITD positive AML cells. Indeed, primary blasts obtained from three patients with *FLT3*-ITD AML treated with OTS514 showed a significant increase in CD11b, (Figure 5G; *P* < 0.01) suggesting an increase in myeloid differentiation.

### TOPK is activated in *FLT3*-ITD AML cells

**Figure 6:**
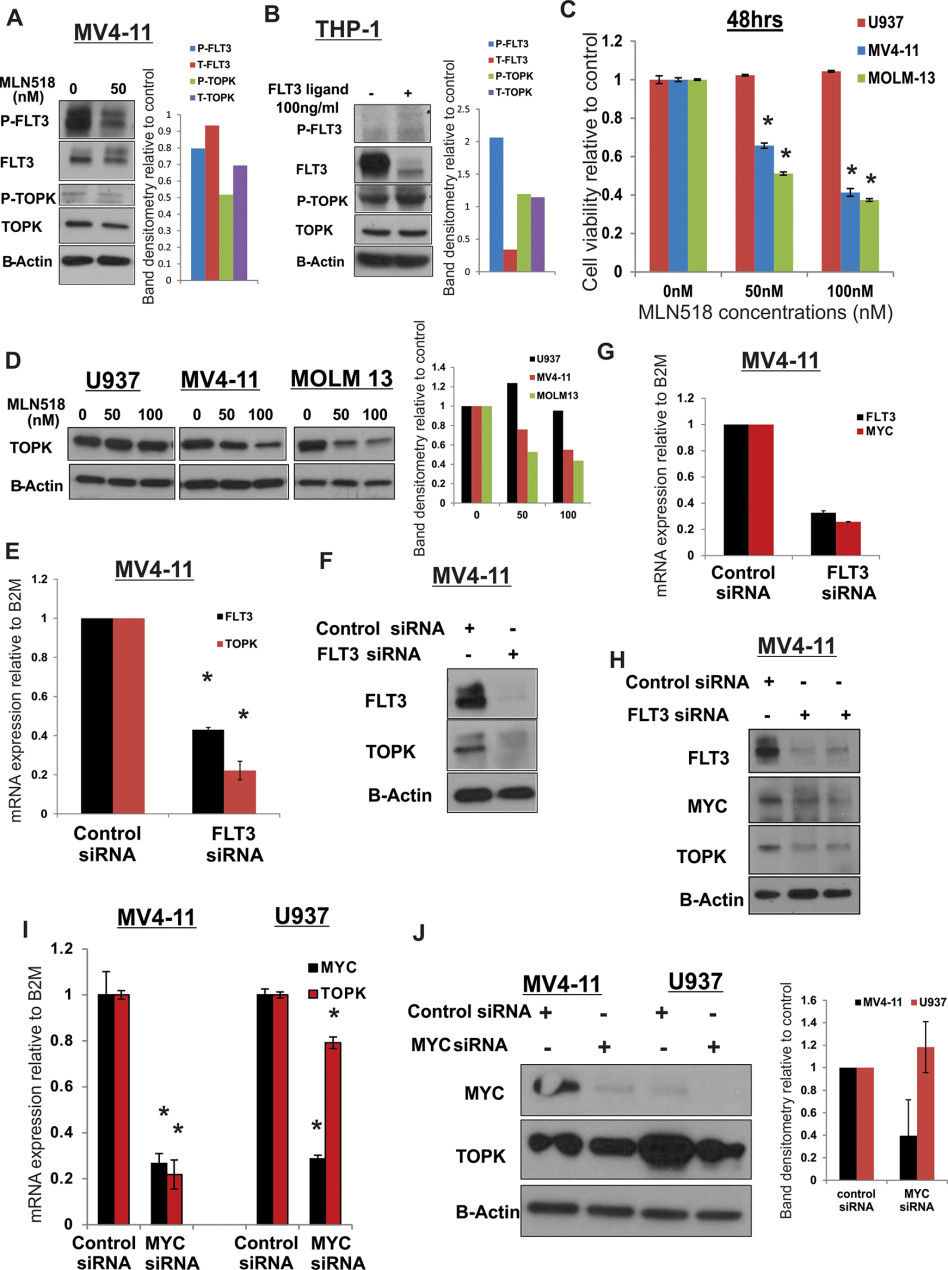
TOPK is activated in *FLT3-ITD* positive in AML cells **A.** MV4-11 cells were treated with 50 nM of FLT3 inhibitor MLN518 for 18 hours and P-FLT3, P-TOPK and total TOPK protein levels were assessed by western blot. **B.** THP-1 cells were treated with 100 ng/ml of FLT3 ligand and P-FLT3, P-TOPK and total TOPK were assessed by western blot. U937, MV4-11 and MOLM13 cells were treated with 50 and 100 nM of FLT3 inhibitor MLN518 **C.** cell viability was assessed by viability assay 48 hours later and **D.** TOPK protein levels were assessed by western blot 18 hours later. MV4-11 cells were transfected with FLT3 siRNA or control siRNA and **E.** TOPK mRNA and **F.** protein levels, **G.** MYC mRNA and **H.** protein levels were assessed. MV4-11 and U937 cells were transfected with *MYC* siRNA or control siRNA and **I.** TOPK mRNA and **J.** protein levels were assessed 48 hours later. Data are presented as Mean ± SEM, *P* values were calculated using Student's *t*-test (*p < 0.05).

Having shown that TOPK inhibition exhibits preferential anti-leukemia activity in AML with *FLT3*-ITD, we speculated that TOPK expression or kinase activity is upregulated in *FLT3*-ITD cells compared to *FLT3*-wt cells. To investigate this hypothesis, we treated MV4-11 cells with 50 nM of FLT3 inhibitor (MLN518) for 18 hours and assessed p-TOPK by western blot. We found a decrease of p-TOPK and t-TOPK in these cells compared to untreated cells (Figure 6A). Furthermore, THP-1 cells (cells that express a high level of FLT3-wt but not FLT3-ITD) treated with FL ligand exhibited a slight increase in the levels of p-TOPK and t-TOPK (Figure 6B). Cells treated with MLN518 for 18 hours at 50 and 100 nM (concentrations that are effective in *FLT3*-ITD mutated cells; Figure 6C) resulted in a significant decrease of total TOPK in *FLT3*-ITD mutated MOLM13 cells, but not in *FLT3*-wt U937 cells (Figure 6D). Additionally, we found that TOPK mRNA and protein levels were significantly decreased following FLT3 knock-down with *FLT3* siRNA in MV4-11 cells (*P* = 0.003; Figure 6E and 6F).

A previous study demonstrated that MYC and E2F1 drive TOPK expression in high-grade malignant lymphoma [45]. MYC is a critical target for CEBPA in granulopoiesis [46] and MYC related genes were found to be upregulated in *FLT3*-ITD compared with *FLT3*-WT AML samples [47–49]. Thus, we speculated that FLT3-ITD upregulates TOPK expression through MYC activity. Indeed MYC expression level decreased significantly in MV4-11 cells transfected with *FLT3*-siRNA in comparison with cells transfected with control-siRNA (Figure 6G and 6H). Furthermore, MYC knockdown in MV4-11 cells resulted in significant decreases of TOPK mRNA and protein levels, less effect was observed in U937 (*P* < 0.001; Figure 6I and 6J).

### CEBPA P30 contributes to TOPK upregulation in AML

**Figure 7:**
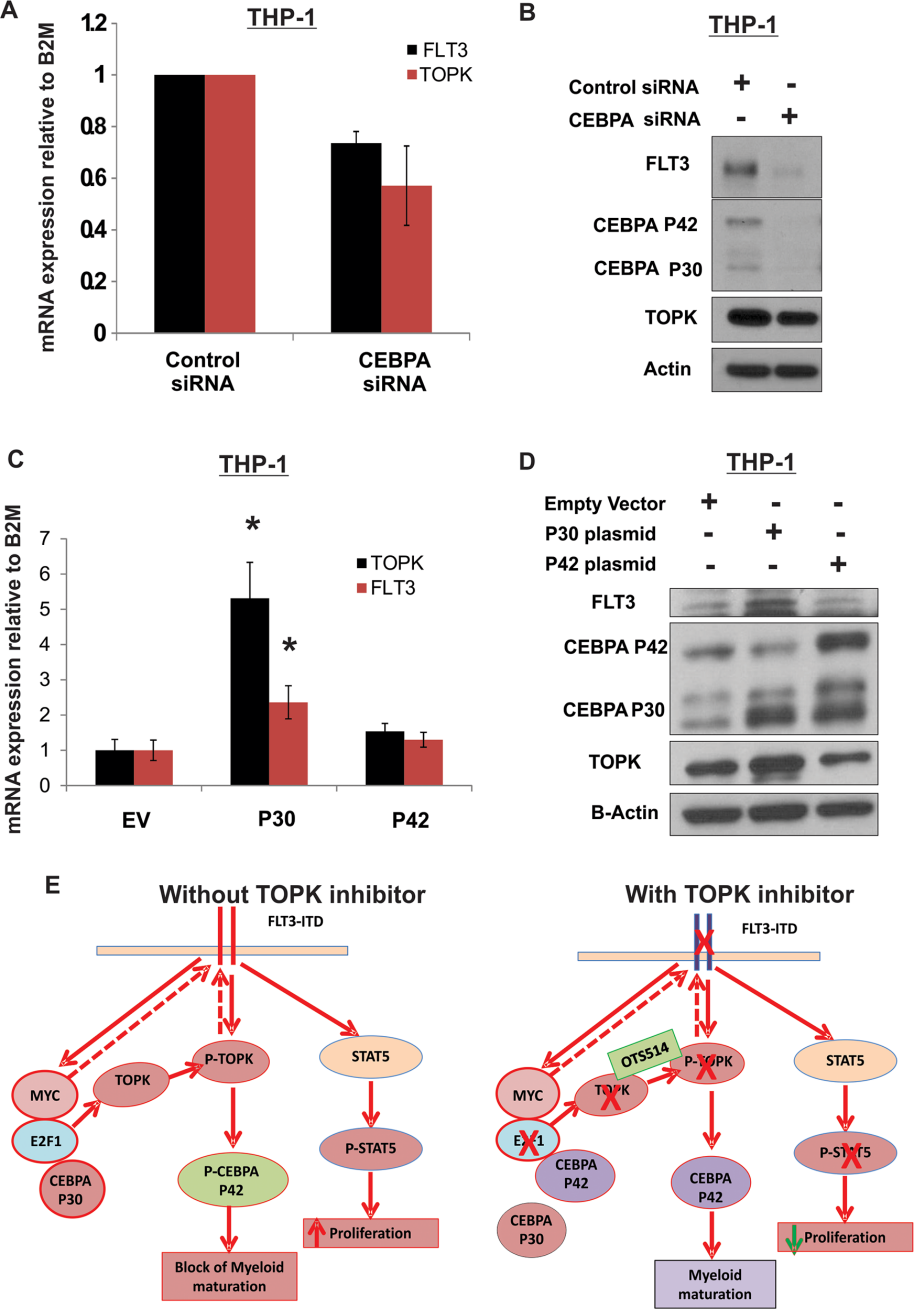
CEBPA P30 contributes to TOPK upregulation in AML THP-1 cells were transfected with *CEBPA* siRNA; **A.** FLT3 and TOPK mRNA and **B.** protein levels were assessed 48 hours later. THP-1 cells transfected with either empty vector: EV, P30 or P42; **C.** TOPK and FLT3 mRNA expression and **D.** protein levels were assessed 48 hours later. **E.** A schematic figure showing the mechanism by which targeting TOPK could affect *FLT3*-ITD positive AML cells. Data are presented as Mean ± SEM, *P* values were calculated using Student's *t*-test (*p < 0.05).

We also examined whether CEBPA contributes to the network that regulates both TOPK and FLT3 expression and explains the reciprocal relationship between the two genes. Interestingly, knock-down of CEBPA in MV4-11 cells did not affect either TOPK or FLT3 expression levels (data are not shown). On the other hand, CEBPA knock-down in THP-1 resulted in a moderate decrease of TOPK and FLT3 mRNA and protein levels (Figure 7A and 7B). CEBPA is expressed as a full-length 42kD protein (P42) and a truncated 30kD isoform protein (P30). The P42 functions as a myeloid differentiation factor that inhibits cell proliferation, whereas the truncated CEBPA(P30) does not have this function [50]. Although P30 could bind E2F, it does not inhibit E2F/MYC transcriptional activity like P42 does [50]. Moreover, mice carrying engineered CEBPA alleles that specifically express the P30 isoform developed AML with complete penetrance [51]. Hence, in order to explain the conflicting results of the CEBPA knock-down experiment, we assessed the contribution of the two CEBPA isoforms on TOPK and FLT3 expression. Here, we utilized a gain of function approach in THP-1 cells. We found that overexpression of P30 resulted in a significant increase in TOPK (~5.3 fold increase, *P* = 0.004) and FLT3 mRNA (~2.3 fold increase, *P* = 0.038) and protein levels; whereas, no significant effect was observed when cells were transfected with P42 (Figure 7C and 7D).

## DISCUSSION

Here we have demonstrated that TOPK is highly expressed in the majority of AML cell lines, and readily detected in myeloblasts from patients with AML in 50% of the cases examined. In contrast, TOPK expression was barely detectable in CD34+ cells derived from healthy donors. These results are consistent with previous data showing that TOPK is upregulated in hematological malignancies [40]. The finding of TOPK overexpression in AML cells suggests that it serves as an important target for drug development in this disease. Indeed, a loss of function approach showed that targeting TOPK in AML cells resulted in a significant decrease of the cell viability and strong induction of apoptosis.

We have recently reported the *in vitro* as well as *in vivo* antitumor effect of the small molecule inhibitor OTS514 which is a highly specific and potent inhibitor for TOPK kinase activity [41]. Here, we investigated the growth-suppressive effect of this compound in AML cells and found that OTS514 has significant anti-leukemia activity in primary blasts but not in CD34+ cells derived from healthy donors. Importantly, OTS514 showed impressive *in vivo* anti-leukemia activity in an aggressive MV4-11 engrafted mouse model, with significant extension of survival in mice treated with this drug compared with the control mice group. In this study we also identified *FLT3*-ITD as a possible predictive molecular marker for TOPK inhibitor therapy. The ability to distinguish a subset of AML patients likely to have a benefit from this therapeutic approach early in preclinical development is important in the design of future clinical trials.

While most of the patients with AML with *FLT3*-ITD achieve a transient remission, they often suffer early relapse with emergence of treatment resistance [2–5]. Currently, allogeneic transplant remains the only curative option for these patients. Several FLT3 inhibitors (sorafenib, midostaurin (PKC-412), quizartinib (AC220), crinolanib, MLN518, CEP-701, SU11248) have been tested in preclinical and clinical studies, and revealed biological activity and acceptable toxicity (15, 16, 31–36), but their impact on clinical outcome remains uncertain [52]. Several mechanisms have been attributed to the insufficient clinical benefit of FLT3 inhibitors including pharmacokinetic challenges and treatment-induced point mutations in the *FLT3* gene that render AML cells resistant to the FLT3 kinase inhibitors [53, 54]. Unlike FLT3 inhibitors currently in clinical trials which target the FLT3 kinase activity, the TOPK inhibitor targets the expression of *FLT3* in *FLT3*-ITD AML cells and, therefore, may overcome the obstacles observed with the FLT3 kinase inhibitors, including the emergence of resistant clones that carry mutations in the kinase domain, or the negative feedback mechanisms resulting in the upregulation of FL ligand and FLT3 expression. Interestingly, we demonstrated that the TOPK inhibitor provides potent anti-leukemia activity, even in primary blasts with the *FLT3*-ITD mutation obtained from patients with AML who progressed after treatment with a potent FLT3 inhibitor (quizartinib, AC220).

Our functional analysis of TOPK identified this kinase as a novel downstream target of FLT3-ITD. TOPK plays an important role in a network involving CEBPA, a crucial transcription factor that is often mutated in AML and responsible for granulocytic differentiation. In *FLT3*-ITD AML, CEBPA differentiation activity is blocked due to its phosphorylation on Ser21 mediated by CDK1 [22]. This functional mechanism may explain the higher sensitivity of FLT3-ITD cells to TOPK inhibition. We have demonstrated that TOPK is another modulator of the CEBPA activity in *FLT3*-ITD mutated AML cells. Previously, p38MAPK and ERK1/2 were also reported to phosphorylate CEBPA on Ser21, inhibit CEBPA activity and granulocyte differentiation in CD34^+^ progenitors [12, 55, 56]. The TOPK inhibitor, however, did not affect p38MAPK or ERK1/2 activities or expression levels (data are not shown), suggesting that the role of TOPK on CEBPA phosphorylation is independent of the ERK and MAPK pathways. Alternative approaches to target *FLT3*-ITD AML cells have focused on modulating pathways that are either downstream of, or interacting with, a FLT3-signaling pathway. Inhibitors of components like ERK1/2, CDK1, STAT5 and PIM1 have been extensively investigated in *FLT3*-ITD AML [10, 22]. In comparison with two other approaches to target *FLT3*-ITD AML using either a FLT3 inhibitor (MLN518) or a CDK1 inhibitor (flavopiridol), OTS514 was significantly more effective in reducing cell viability at a lower IC50 than MLN418 and flavopiridol (Supplementary Figure S9).

In conclusion, our study demonstrates that TOPK is highly expressed in AML cell lines and in primary leukemia cells from patients with AML, and serves as a novel therapeutic target. Targeting TOPK with OTS514 showed a remarkable growth-suppressive effect *in vitro* as well as *in vivo* in *FLT3*-ITD AML cells. This preferential activity of OTS514 in *FLT3*-ITD is likely due to the strong reduction of FLT3 protein expression and the resulting downregulation of its downstream targets (i.e., STAT5) in addition to the decrease of CEBPA phosphorylation resulting from TOPK inhibition. Importantly, we identify a network mechanism by which TOPK is deregulated in AML involving both FLT3 and CEBPA, both of which play a central role in leukemogenesis. Inhibition of TOPK decreases CEBPA P42 phosphorylation which then becomes available to bind E2F1 and inhibits E2F1/MYC transcriptional activity resulting in the decrease of TOPK expression, and possibly FLT3 (Figure 7E). Our proposed mechanistic FLT3/TOPK/CEBPA network identifies TOPK as a valid therapeutic target in AML. Thus, targeting TOPK represents a novel therapeutic approach particularly for patients with adverse risk *FLT3*-ITD AML.

## MATERIALS AND METHODS

### Cell lines and primary blasts for *in vitro* experiments

AML cell lines (ATCC, Manassas, VA) were cultured in RPMI medium supplemented with 10–20% fetal bovine serum (FBS) (Life Technologies, Grand Island, NY). Myeloblasts from patients with AML were maintained in RPMI medium supplemented with 20% FBS, and 1x StemSpan CC100 (StemCell Technologies, Vancouver, Canada). Leukemia cells were obtained by apheresis of blood or bone marrow samples collected from patients treated at the University of Chicago (UC) and stored in the UC Leukemia Tissue Bank. Informed consent to use the tissue for investigational studies was obtained from each patient according to UC institutional guidelines.

### Transient transfection, RNA interference

Transient transfection of cells was performed utilizing 1 nmol (the final concentration in the medium was unclear) of siRNA and 100 ul Gene Pulser buffer per reaction, and the cells were electroporated using the Bio-Rad Gene Pulse Xcell (Bio-Rad, Hercules, CA) or Amaxa Nucleofector Kit (Lonza, Basel Switzerland) according to manufacturer's instructions.

### RNA extraction, RNA expression quantification

Total RNA was extracted using Trizol reagent (Life Technologies). *TOPK, FLT3, MYC* mRNA expression in AML cells was measured using the ViiA 7 system according to the manufacturer's instructions. Each cDNA was synthesized using SuperScript III reagents (Life Technologies,) according to the manufacturer's instructions. Quantitative Real-Time PCR (qRT-PCR) was performed using commercially available TaqMan Gene Expression Assay primers and probes with the ViiA 7 system (Life Technologies). The expression levels were normalized to *B2M* gene.

### Western blot and immunoprecipitation analyses and antibodies

Western-blot and immunoprecipitation analyses were performed as previously described [42]. A list of the antibodies used in the study is provided in the supplemental material.

### Clonogenic and viability analyses

Methylcellulose clonogenic assays were carried out by plating 2 × 10^4^ primary blasts in 0.9% MethoCult (StemCell Technologies, Vancouver, Canada) [57]. Colony numbers were scored 10 days later, by counting all colonies per well. For viability analysis, cell cycle count assay was performed in a 96-well plate and 5 × 10^4^ cells were plated per well. Cell counting kit-8 (CCK-8 assay; Dojindo Molecular Technologies, Inc., Kumamoto, Japan) was used for the reaction.

For viability and apoptosis analysis, cells were collected, spun down, then washed with PBS and re-suspended in 50 μl binding buffer containing 2 μL of Annexin V (eBioscience, San Diego, CA), and 5 μL propidium iodide (PI) (eBioscience). After 20 min incubation, fluorescence was quantified by flow cytometry on a FACSCalibur instrument [42].

### Immunofluorescent staining, flow cytometry

Cells were washed with PBS, spun down and stained with CD11b antibody (eBioscience, San Diego, CA) with 20-min incubation at room temperature; cells were then washed with PBS and re-suspended in PBS. Fluorescence was quantified by flow cytometry on a FACSCalibur instrument [58].

### *In vivo* studies

To investigate the anti-leukemia activity of OTS514 *in vivo*, we utilized a previously reported *FLT3*-ITD engraft murine model and detailed in the supplemental materials [42].

### Statistical analysis

Mechanistic and biological experiments were analyzed with paired and unpaired two-sided *t*-tests. *P* values < .05 were considered statistically significant. Experiments were performed in triplicate (except for when patient blasts were used, these experiment were done in duplicate when possible), results were presented by Mean ± SEM. Percent survival were calculated using Kaplan-Meier method and the Log-rank test was used to compare the survival curves.

## SUPPLEMENTARY MATERIALS AND METHODS FIGURES AND TABLES


