# Supplementary material for: T-LAK cell-originated protein kinase presents a novel therapeutic target in FLT3-ITD mutated acute myeloid leukemia
Source: Oncotarget. 2015 Oct 2;6(32):33410–25. doi: 10.18632/oncotarget.5418 (PMC4741775; doi:10.18632/oncotarget.5418)
Supplement: Supplementary file 1 [file oncotarget-06-33410-s001.pdf]

SUPPLEMENTARY MATERIALS AND METHODS

List of antibodies used in the study

Antibody	Company	Mouse/Rabbit	#
Flt-3/FIk-2 (sc-18) sc-480	Santa Cruz Biotechnology	Rabbit Polyclonal	F1713
P-Flt3 (Y589/591) 30D4	Cell Signaling Technology	Rabbit	3464S
C/EBPalpha (D56F10) XP®	Cell Signaling Technology	Rabbit	8178S
P-C/EBPalpha (S21)	Cell Signaling Technology	Rabbit	2841S
Purified Mouse Anti-PBK	BD Biosciences	Mouse	612170
P-PBK/TOPK (T9)	Cell Signaling Technology	Rabbit	4941S
p38 MAPK (D13E1) XP®	Cell Signaling Technology	Rabbit	8690S
P-p38 MAPK (T180/Y182) 28B10	Cell Signaling Technology	Mouse	9216S
p44/42 MAPK (Erk1/2) (137F5)	Cell Signaling Technology	Rabbit	4695S
P-p44/42 MAPK (T202/Y204)	Cell Signaling Technology	Rabbit	9101S
cdc2 (POH1)	Cell Signaling Technology	Mouse	9116S
P-cdc2 (Y15)	Cell Signaling Technology	Rabbit	9111S
Stat5	Cell Signaling Technology	Rabbit	9363S
P-Stat5 (Y694)	Cell Signaling Technology	Rabbit	9351S
Monoclonal Anti -B-Actin	SIGMA Life Science	Mouse	122M4782
Cyclin B1 (V152)	Cell Signaling Technology	Mouse	4135S
P-Cyclin B1 (S147)	Cell Signaling Technology	Rabbit	4131S
P-Histone H3 (Ser10) 6G3	Cell Signaling Technology	Mouse	9706S

Reagents

siRNA sequence targeting TOPK was as follows: *TOPK* siRNAs sense sequence (GUGUGGCUUGCGUAAAUAA), antisense sequence (UUAUUUACGCAAGCCACAC) (1) and siRNA targeting MYC were SASI_Hs01_00222676 and SASI_Hs01_00222677 (Sigma-Aldrich, St. Louis, MO). L-003137–00-0005 On target plus Human FLT3 (2322) siRNA- SMART pool was used to target FLT3 (Dharmacon, Schwerte, Germany); CEBPA (ID 1050) Trilencer-27 Human siRNA to target CEBPA (Origene, Rockville, MD). OTS514 was provided by Oncotherapy Science (Kawasaki, Japan).

Cell cycle analysis

Cells were treated with 50 nM of OTS514, 24 hours later cells were incubated with Brdu for 45 minutes, then collected, washed with PBS, fixed and stained according to BD Pharmingen FITC Brdu Flow kit instruction (BD, San Jose, CA). Samples were then analyzed by flow cytometry on a FACSCalibur instrument (2).

In vitro kinase assay

Recombinant TOPK protein or recombinant FLT3 protein (2 ul of 100 ng) were incubated at room temperature (RT) with 1 ul of increasing concentrations of OTS514 for 10 minutes. Then 2 ul mix of ATP (25 uM) and MBP substrate (0.5 ug) in kinase buffer were added to the previous reaction mix and mixed well for 2 minutes then incubated at RT for 60 minutes. Kinase activity was measured using ADP-Glo kinase enzyme system protocol (Promega, Madison, WI).

In vivo studies

To investigate the anti-leukemia activity of OTS514 *in vivo*, we utilized a previously reported *FLT3*-ITD engraft murine model(3). Briefly, 7 ~ 8 week-old non-obese diabetic severe combined immunodeficient gamma (NSG) mice (NOD.Cg-Prkdcscid Il2rgtm1Wjl/SzJ, The Jackson Laboratory, Bar Harbor, ME) were intravenously (IV) injected via tail vein with 1×10^5 cells/mouse spleen

MNC from 5th generation transplanted NSG mice (3rd generation transplanted cells were kindly provided by Ramasamy Santhanam and Guido Marcucci. Engraftment of cells was previously assessed by white blood cell count (WBC) and huCD45 staining using flow cytometry. Using this model, leukemia will develop at only two weeks from injection and the median survival of these mice is approximately 4 weeks(3). All of the experiments were conducted in accordance with the institutional guidelines for animal care and use at University of Chicago and OncoTherapy Science. For pharmacodynamics study, five mice were transplanted with (1.0×10^5) spleen cells obtained from MV4-11 transplanted NSG mice. Starting on day 13 after engraftment, mice were treated with OTS514 (N = 3, 7.5mg/kg IV) or vehicle (N = 2, same volume IV) daily for 4 days. Mice were sacrificed 24 hours after last dose, and spleens were obtained and weighed. For survival analysis six mice were used per group and 3 mice were used as negative controls (no leukemia and no treatment). Ten days after engraftment, WBC count was assessed to confirm engraftment. OTS514 (7.5 mg/kg) or vehicle (same volume), was given by IV administration by tail vein daily for 3 weeks starting 10 days after engraftment, or until euthanasia criteria were met. Expected median survival of untreated animals in this model is 28 days. Mice were weighed daily and checked for signs of dehydration, discomfort or toxicity. On the day of administration, doses were recalculated for each animal after weighing to maintain 7.5 mg/kg.

REFERENCES

1. Park JH, Nishidate T, Nakamura Y, Katagiri T. Critical roles of T-LAK cell-originated protein kinase in cytokinesis. Cancer science. 2010; 101:403–11. PubMed PMID: 19900192.
2. Alachkar H, Mutonga MB, Metzeler KH, Fulton N, Malnassy G, Herold T, et al. Preclinical efficacy of maternal embryonic leucine-zipper kinase (MELK) inhibition in acute myeloid leukemia. Oncotarget. 2014; PubMed PMID: 25365263.
3. Alachkar H, Santhanam R, Harb JG, Lucas DM, Oaks JJ, Hickey CJ, et al. Silvestrol exhibits significant *in vivo* and *in vitro* antileukemic activities and inhibits FLT3 and miR-155 expressions in acute myeloid leukemia. Journal of hematology & oncology. 2013; 6:21. PubMed PMID: 23497456. PubMed PMID: 3623627002.

SUPPLEMENTARY FIGURES AND TABLES

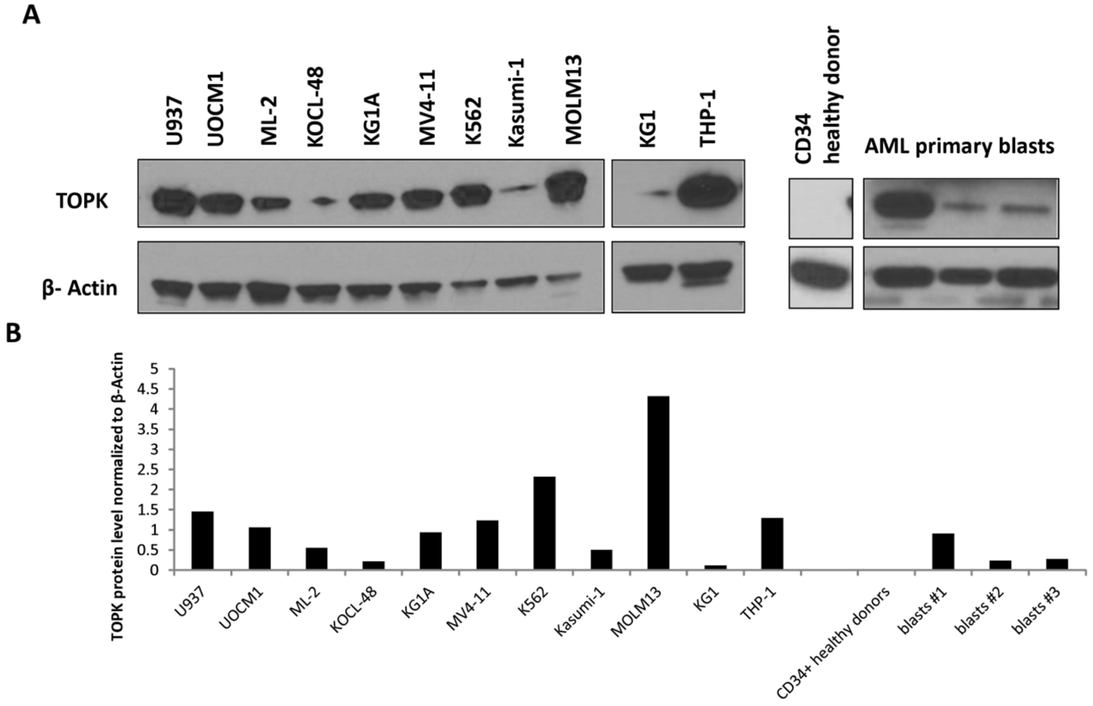

Supplementary Figure S1: TOPK is expressed in AML cell lines and primary blasts. A. TOPK protein level was evaluated in AML cell lines, CD34+ cells obtained from healthy donor and AML primary blasts, and **B.** a quantification of protein band intensity is depicted.

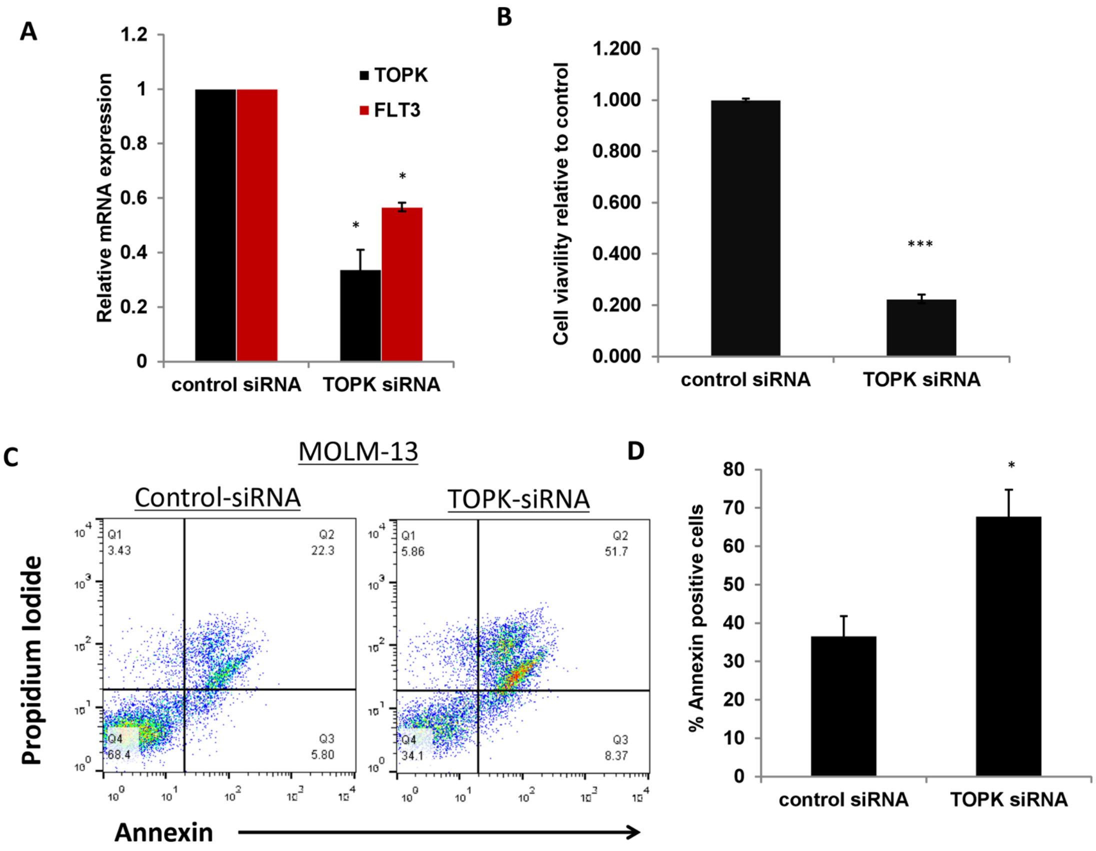

Supplementary Figure S2: TOPK Knock-down decreases cell viability and induces apoptosis in MOLM13 cells. MOLM13 cells were transfected with TOPK siRNA or control siRNA; **A.** Relative mRNA expression of TOPK and FLT3. **B.** Viability assay were performed 48 hours following transfection. **C.** A representative result of apoptosis assay performed using annexin V and PI staining in MOLM13 cells 48 hours following transfection with TOPK siRNA. **D.** Quantification analysis of percentage of annexin V positive cells, data are obtained from three independent experiments.

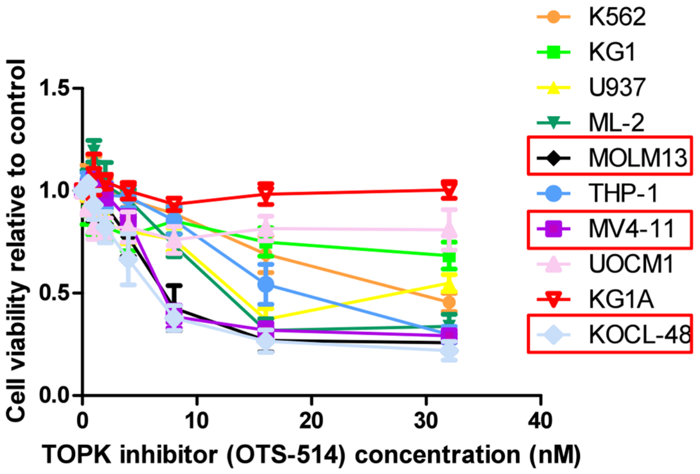

Cell Line	IC50
K562	36.76
KG1	48.69
U937	19.15
ML-2	17.85
MOLM13	9.025
THP-1	23.52
MV4-11	10.7
UOCM1	71.01
KG1A	2617
KOCL-48	7.069

Supplementary Figure S3: TOPK inhibitor exhibits preferential anti-leukemia activity in *FLT3*-ITD AML. AML cell lines (n = 10) were treated with increasing concentration of TOPK inhibitor OTS514, and CCK-8 assay was performed 48 hours post-treatment.

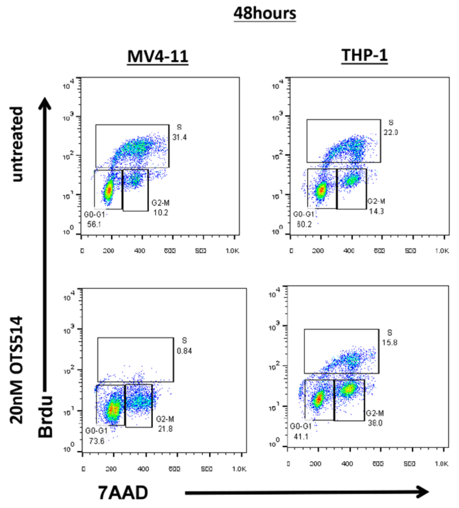

Supplementary Figure S4: TOPK inhibitor exhibits preferential anti-leukemia activity in *FLT3*-ITD AML. MV4-11 and THP-1 cells were treated with 20 nM OTS514 and cell cycle analysis was performed using Brdu and 7AAD staining 48 hours later.

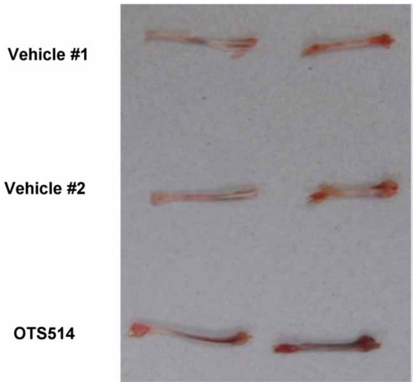

Supplementary Figure S5: Bones of mouse treated with OTS514. One mouse died after the 19th dose of OTS514 (7.5 mg/kg IV per day), bone marrow on the mouse looked normal in comparison with two vehicle treated mice died on the same day.

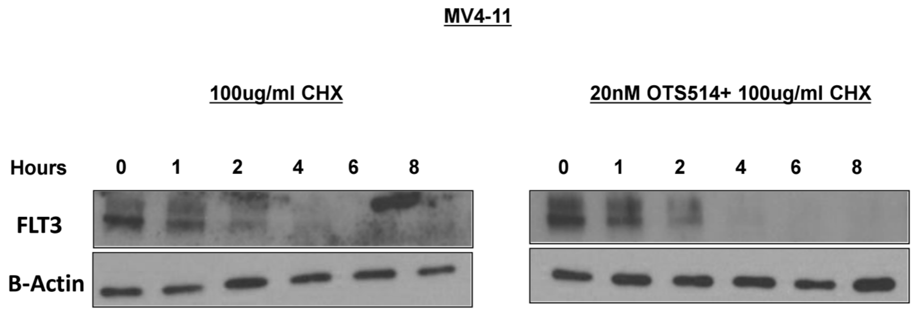

Supplementary Figure S6: TOPK inhibitor OTS514 does not affect FLT3 protein stability in MV4-11 cells. MV4-11 cells were treated with 100 µg/ml cyclohexamide and 20 nMTOPK inhibitor OTS514 and FLT3 protein degradation over time was measured by western blot, and compared to that of cells treated with cyclohexamide alone.

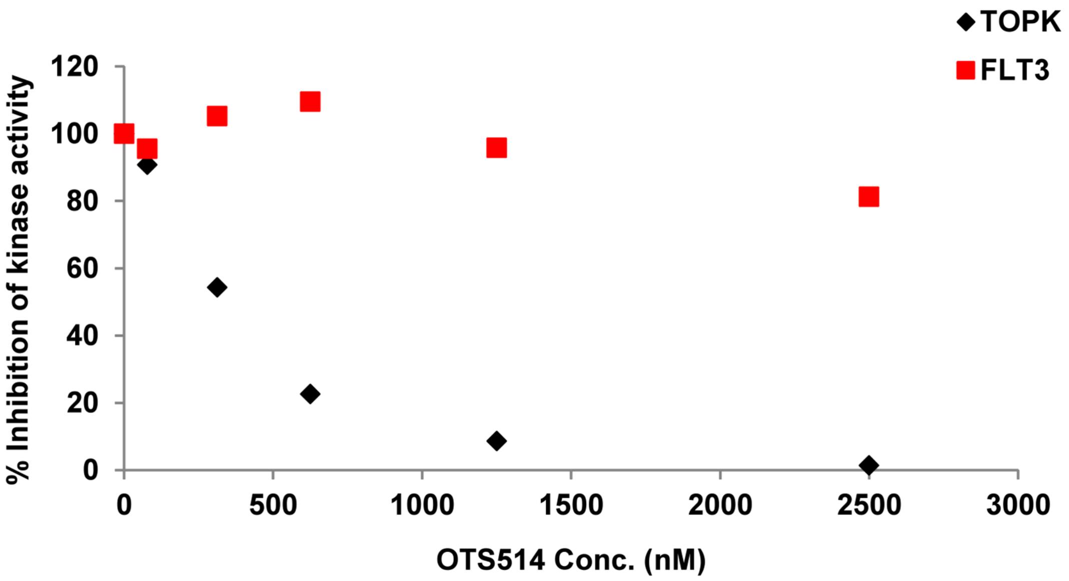

Supplementary Figure S7: TOPK inhibitor OTS514 inhibits TOPK but not FLT3 kinase activity. TOPK or FLT3 recombinant proteins were incubated with increasing concentration of OTS514 and kinase assay was performed. Inhibition of the protein kinase activity was assessed.

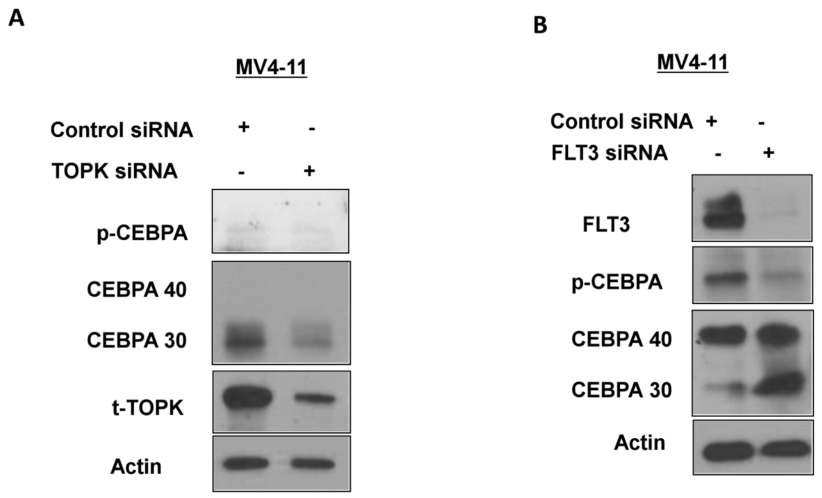

Supplementary Figure S8: TOPK affects p-CEBPA levels in MV4-11 cells. MV4-11 cells were transfected with TOPK siRNA **A.** or FLT3 siRNA **B.** and p-CEBPA levels were measure by western blot.

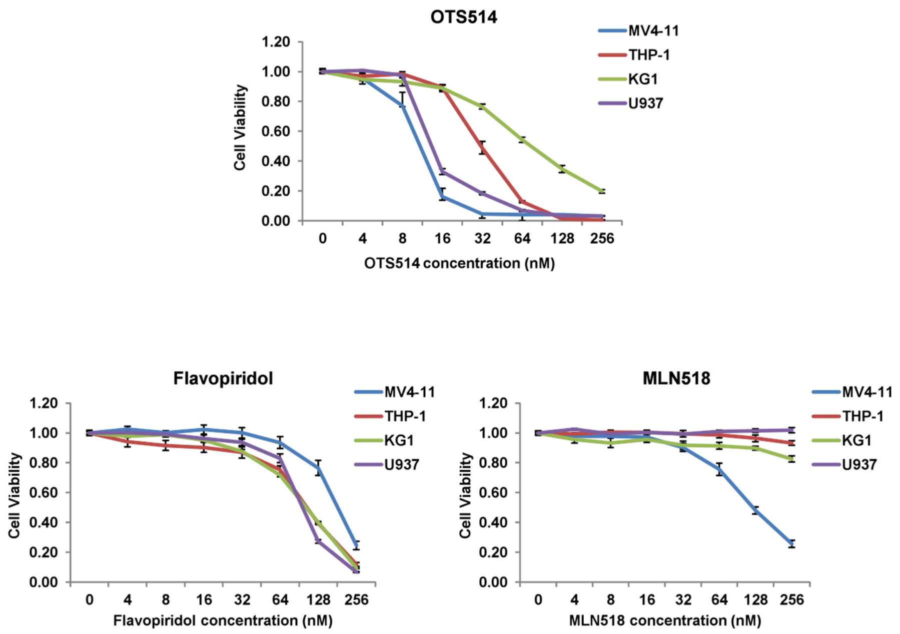

Supplementary Figure S9: OTS514 is superior to CDK1 and FLT3 inhibitors in *FLT3*-ITD AML cells. MV4-11, THP-1, KG1 and U937 cells were treated with TOPK inhibitor (OTS514), CDK1 inhibitor (Flavopiridol), or FLT3 inhibitor (MLN518), and CCK-8 assay was performed 48 hours later to assess cell viability.

Supplementary Table S1: List of cell lines and their cytogenetic and molecular aberrations including their FLT3 mutational status

Cell Line	Cytogenetic and molecular aberrations
K562	BCR-ABL, FLT3-wt
KG1	FLT3-wt
U937	FLT3-wt
ML-2	FLT3-wt
MOLM13	t(9;11), FLT3-ITD
THP-1	FLT3-wt
MV4-11	t(4;11), FLT3-ITD
UOCM1	del (5), –7, FLT3-wt
KG1A	FLT3-wt
KOCL-48	t(4;11), *FLT3*-D835
Kasumi-1	t(8;21), Kit mutation

Supplementary Table S2: List of patient's samples and their FLT3 mutational status

patient number	FLT3 mutation status
Pt #1	FLT3-ITD
Pt #2	FLT3-WT
Pt #3	FLT3-WT
Pt #4	FLT3-WT
Pt #5	FLT3-ITD
Pt #6	FLT3-ITD
Pt #7	FLT3-ITD
